# Meeting abstracts from the 64th British Thyroid Association Annual Meeting: Newcastle, UK. 13/05/2016

**DOI:** 10.1186/s13044-016-0036-8

**Published:** 2017-02-13

**Authors:** Luigi Bartalena, Eric Fliers, Nicola Hellen, Peter N. Taylor, Arron Lacey, Daniel Thayer, Mohd Draman Yusof, Arshiya Tabasum, Illaria Muller, Luke Marsh, Marian Ludgate, Alex Rees, Kristien Boelaert, Shiao Chan, Scott Nelson, Aled Rees, John H. Lazarus, Colin M. Dayan, Bijay Vaidya, Onyebuchi Okosieme, Vikki L. Poole, Alice Fletcher, Bhavika Modasia, Neil Sharma, Rebecca J. Thompson, Waraporn Imruetaicharoenchoke, Martin L. Read, Kristien Boelaert, Christopher J. McCabe, Vicki E. Smith, Martin L. Read, Jim C. W. Fong, Waraporn Imruetaicharoenchoke, Bhavika Modasia, Hannah Nieto, Alice Fletcher, Rebecca Thompson, Neil Sharma, John C. Watkinson, Andrew S. Turnell, Kristien Boelaert, Vicki E. Smith, Christopher J. McCabe, Nadia Pariani, Mark Willis, Ilaria Muller, Sarah Healy, Taha Nasser, Joanne Jones, Krishna Chatterjee, Colin Dayan, Neil Robertson, Alasdair Coles, Carla Moran, Charlotte Hales, Peter Taylor, Sue Channon, Kirsten McEwan, Aled Rees, John Gregory, Ilaria Muller, Mohd S. Draman, Colin Dayan, Kate Langley, Anita Thapar, John Lazarus, Marian Ludgate, Barbara Torlinska, Jayne Franklyn, Jamie Coleman, Kristien Boelaert, Charlotte Hales, Peter Taylor, Sue Channon, Kirsten McEwan, Lei Zhang, Ameen Bakhsh, Michael Gyedu, Aled Rees, Onyebuchi Okosieme, John Gregory, Ilaria Muller, Mohd S. Draman, Colin Dayan, John Lazarus, Marian Ludgate, Enrique Soto-Pedre, Graham P. Leese, Kaixin Zhou, Martin L. Read, Joel A. Smith, Jon Hoffman, Vicki E. Smith, Neil V. Morgan, Christopher Campbell, Naomi C. Wake, John C. Watkinson, Yvonne Wallis, Eamonn R. Maher, Christopher J. McCabe, Emma R. Woodward, Su A. Tee, David R. Bishop, Ahmed Al-Sharefi, Owain Leng, Robert A. James, Rashim Salota, Andrew Rodin, Nikhil Johri, Stephen L. Hyer, Thomas Mcdonnell, Cheong G. Ooi, Bridget A Knight, Beverley M. Shields, Elizabeth Pearce, Lewis Braverman, Xuemei He, Rachel Sturley, Bijay Vaidya

**Affiliations:** 1 Endocrine Unit, University of Insubria, Varese, Italy; 2 Department of Endocrinology and Metabolism at the Academic Medical Center, University of Amsterdam, Amsterdam, Netherlands; 3 National Heart and Lung Institute, Imperial College, London, UK; 4 Thyroid Research Group, School of Medicine, Cardiff University, Cardiff, UK; 5 Secure Anonymised Information Linkage (SAIL) Databank, Swansea University, Swansea, UK; 6 Obstetrics and Gynaecology Department, University Hospital Wales, Cardiff, UK; 7 University of Birmingham and Queen Elizabeth Hospital, Birmingham, UK; 8 Department of Obstetrics and Gynaecology, National University of Singapore, Singapore, Singapore; 9 Obstetrics & Gynaecology Department, University of Glasgow, Glasgow, UK; 10 Department of Endocrinology, Royal Devon and Exeter NHS Foundation Trust, Exeter, UK; 11 Institute of Metabolism and Systems Research, University of Birmingham, Birmingham, UK; 12 Institute of Metabolism and Systems Research, University of Birmingham, Birmingham, UK; 13 University Hospitals Birmingham National Health Service Foundation Trust, Birmingham, UK; 14 Institute of Cancer and Genomic Sciences, University of Birmingham, Birmingham, UK; 15 Wellcome-MRC Institute of Metabolic Science, University of Cambridge, Cambridge, UK; 16 Institute of Psychological Medicine and Clinical Neuroscience, Cardiff University & University Hospital of Wales, Cardiff, UK; 17 Department & Institute of Molecular & Experimental Medicine, Cardiff University, Cardiff, UK; 18 Department of Clinical Neurosciences, University of Cambridge, Cambridge, UK; 19 Thyroid Research Group, Cardiff University School of Medicine, Cardiff, UK; 20 Institute of Primary Care & Public Health, Cardiff University School of Medicine, Cardiff, UK; 21 Institute of Psychological Medicine and Clinical Neurosciences, Cardiff University School of Medicine, Cardiff, UK; 22 Institute of Metabolism and Systems Research, University of Birmingham, Birmingham, UK; 23 Institute of Clinical Sciences, University of Birmingham, Birmingham, UK; 24 Thyroid Research Group, Cardiff University School of Medicine, Cardiff, UK; 25 Institute of Primary Care & Public Health, Cardiff University School of Medicine, Cardiff, UK; 26 Division of Molecular and Clinical Medicine, School of Medicine, Ninewells Hospital and Medical School, University of Dundee, Dundee, Scotland UK; 27 Department of Endocrinology and Diabetes, Ninewells Hospital and Medical School, University of Dundee, Dundee, Scotland UK; 28 Institute of Metabolism and Systems Research, University of Birmingham, Birmingham, UK; 29 West Midlands Regional Genetics Service, Birmingham Women’s Hospital, Birmingham, UK; 30 Institute of Cardiovascular Sciences, University of Birmingham, Birmingham, UK; 31 University Hospitals Birmingham National Health Service Foundation Trust, Birmingham, UK; 32 West Midlands Regional Genetics Laboratory, Birmingham Women’s Hospital, Birmingham, UK; 33 Department of Medical Genetics, University of Cambridge, Cambridge, UK; 34 Manchester Centre for Genomic Medicine, St. Mary’s Hospital, Manchester, UK; 35 Department of Diabetes and Endocrinology, Northumbria Healthcare NHS Foundation Trust, Newcastle, UK; 36 Department of Diabetes and Endocrinology, Sunderland Royal Hospital, Sunderland, UK; 37 Department of Diabetes and Endocrinology, Darlington Memorial Hospital, Darlington, UK; 38 Department of Diabetes and Endocrinology, South Tyneside General Hospital, South Tyneside, UK; 39 Department of Endocrinology, Royal Victoria Infirmary, Newcastle, UK; 40 Chemical Pathology, Epsom and St Helier Hospitals NHS Trust, Carshalton, SM5 1AA UK; 41 Departments of Endocrinology, Epsom and St Helier Hospitals NHS Trust, Carshalton, SM5 1AA UK; 42 Department of Diabetes and Endocrinology, Aintree University Hospital, Liverpool, UK; 43 NIHR Exeter Clinical Research Facility, University of Exeter Medical School, Exeter, UK; 44 R&D Department, Royal Devon and Exeter NHS Foundation Trust, Exeter, UK; 45 Section of Endocrinology, Diabetes & Nutrition, Boston University School of Medicine, Boston, MA USA; 46 Department of Women’s Health, Royal Devon and Exeter NHS Foundation Trust, Exeter, UK; 47 Department of Endocrinology, Royal Devon and Exeter NHS Foundation Trust, Exeter, UK

## S1 Clinical Features and Evidence-Based Management of Graves’ Orbitopathy

### Luigi Bartalena (luigi.bartalena@uninsubria.it)

#### Endocrine Unit, University of Insubria, Varese, Italy

Graves’ orbitopathy (GO) is the main extrathyroidal manifestation of Graves’ disease. When fully expressed, it is characterized by inflammatory soft tissue changes, exophthalmos, ocular dysmotility causing diplopia, and, rarely, sight-threatening dysthyroid optic neuropathy (DON). The prevalence of GO among Graves’ patients seems lately declining, probably due to early diagnosis, early intervention on risk factors associated with its occurrence or progression (smoking, uncontrolled thyroid dysfunction), early correction of hyper and hypothyroidism. Only about 25–30% of newly diagnosed Graves’ hyperthyroids are affected with GO, which is usually mild and rarely progressive. Assessment of activity and severity of GO according to standardized criteria is fundamental to plan management. The European Thyroid Association and the European Group on Graves’ Orbitopathy (EUGOGO) have recently published the first guideline on management of GO. Mild GO usually requires only a watchful strategy, in addition to local measures (eye drops, ointments) and removal of risk factors. Intravenous glucocorticoids (ivGCs) are the first-line treatment for moderate-to-severe and active GO, as demonstrated by randomized clinical trials. When ivGCs fail or GO recurs after treatment withdrawal, options include a second course of ivGCs, oral GCs combined with orbital radiotherapy or cyclosporine, rituximab. Evidence that the any of the above treatment be effective in the context of a poor response to a first course of ivGCs is limited and should be investigated in larger studies. In addition to rituximab, ongoing investigations are exploring the role of other biologics targeting, e.g., the IGF-1 receptor or the IL-6 receptor, and results will probably available in 1–2 years. When GO has been treated medically and is inactive, rehabilitative surgery (orbital decompression, squint surgery, eyelid surgery) is often needed.

## S2 Role of T3 and TRH in the hypothalamic regulation of energy metabolism

### Eric Fliers (e.fliers@amc.uva.nl)

#### Department of Endocrinology and Metabolism at the Academic Medical Center, University of Amsterdam, Amsterdam, Netherlands

The relation between thyrotoxicosis, the clinical syndrome resulting from exposure to excessive thyroid hormone, and the autonomic nervous system remains enigmatic. Recent experiments from our lab and others have shown that T3 may act within several hypothalamic nuclei to modulate hepatic glucose metabolism and brown adipose tissue (BAT) activity. These effects are mediated via pre-autonomic neurons connecting the paraventricular and ventromedial nuclei (PVN and VMH) with these peripheral organs via neural pathways. Intrahypothalamic effects of T3 on glucose metabolism in the liver could be modulated by selective hepatic sympathetic and parasympathetic denervation. Thyroid hormone appeared to stimulate hepatic glucose production via a sympathetic pathway, representing a novel central route for thyroid hormone action. As most of the experiments were performed in a rather acute setting we recently developed a model for chronic intrahypothalamic T3 administration in rats, allowing for the assessment of chronic metabolic effects of intrahypothalamic T3 administration. In addition to T3, we now investigate the role of intrahypothalamic TRH in energy metabolism. Further elucidation of intrahypothalamic effects of thyroid hormones on autonomic outflow to metabolic organs will add to our understanding of the metabolic effects of hyperthyroidism.

## S3 Triiodothyronine, Deiodinase 3 and Stem Cell-derived Cardiomyocyte Maturation

### Nicola Hellen (n.hellen@imperial.ac.uk)

#### National Heart and Lung institute, Imperial College, London, UK

Functional cardiomyocytes can be derived from both human embryonic (hESCs) and induced pluripotent (hiPSCs) stem cells. These pluripotent stem cell-derived cardiomyocytes (hPSC-CM) hold great potential for regenerative medicine and *in vitro* screening. However, the phenotype is immature and more closely resembles that of foetal/neonatal cardiomyocytes. In the adult heart thyroid hormone has pleiotropic effects on contractility and energy metabolism. The active form of thyroid hormone, triiodothyronine (T3), is known to play a role during foetal cardiomyocyte maturation and has recently been shown to promote cardiac differentiation and enhance hPSC-CM maturation *in vitro*. During development the enzyme, deiodinase 3 (D3), protects the foetus against maternal thyroid hormone by metabolism of T3 into inactive products. Down regulation of D3 at appropriate times during development allows physiological growth and maturation of cardiomyocytes in response to thyroid hormone. We show that stimulation of the thyroid hormone pathway can enhance the maturation of hPSC-CM and that further investigation into the contribution of individual components of this pathway is warranted.

## O1 Controlled Antenatal Thyroid Study: Obstetric Outcomes

### Peter N Taylor^1^, Arron Lacey^2^, Daniel Thayer^2^, Mohd Draman Yusof^1^, Arshiya Tabasum^1^, Illaria Muller^1^, Luke Marsh^1^, Marian Ludgate^1^, Alex Rees^3^, Kristien Boelaert^4^, Shiao Chan^5^, Scott Nelson^6^, Aled Rees^1^, John H Lazarus^1^, Colin M Dayan^1^, Bijay Vaidya^7^, Onyebuchi Okosieme^1^

#### ^1^Thyroid Research Group, School of Medicine, Cardiff University, Cardiff, UK; ^2^Secure Anonymised Information Linkage (SAIL) Databank, Swansea University, Swansea, UK; ^3^Obstetrics and Gynaecology Department, University Hospital Wales, Cardiff, UK; ^4^University of Birmingham and Queen Elizabeth Hospital, Birmingham, UK; ^5^Department of Obstetrics and Gynaecology, National University of Singapore, Singapore, Singapore; ^6^Obstetrics & Gynaecology Department, University of Glasgow, Glasgow, UK; ^7^Department of Endocrinology, Royal Devon and Exeter NHS Foundation Trust, Exeter, UK

##### **Correspondence:** Peter N Taylor (TaylorPN@Cardiff.ac.uk)

**Background**

Suboptimal thyroid function in pregnancy is associated with adverse obstetric outcomes but it is unclear whether levothyroxine treatment, initiated during pregnancy is beneficial. We investigated whether correction of abnormal thyroid function during pregnancy is associated with improved obstetric outcomes.

**Methods**

Retrospective analysis of the Controlled Antenatal Thyroid Screening (CATS) study with obstetric outcomes obtained through data-linkage in the Secure Anonymised Information Linkage (SAIL) databank. Setting: Welsh participants from CATS. Participants: 13,506 pregnant women; 12,874 women had normal thyroid function, 320 had subclinical hypothyroidism (SCH), 281 had isolated hypothyroxinemia (IH) and 31 had overt hypothyroidism. Main Outcome Measures: Odds of stillbirths (fetal demise after 24 weeks gestation), Caesarean sections, prematurity and abnormal birth-weight by thyroid and treatment status.

**Results**

Untreated women with SCH had increased odds of stillbirth compared to women with normal thyroid function OR = 5.73 (95%CI 1.74, 18.9) p = 0.003. No stillbirths occurred in women receiving levothyroxine. In analysis of women with IH, untreated women had an increased risk of early (≤37 weeks) caesarean section than those who received levothyroxine (6% vs 0%) p = 0.006. Untreated women with IH also had earlier mean gestational age 38.8 (SD 2.34) weeks vs. 39.7 (SD 1.94) weeks p = 0.002 and lower mean birth-weight 3353 g (SD 639) vs. 3558 g (SD 532) p = 0.004.

**Conclusion**

Both SCH and IH are associated with adverse obstetric outcomes. In IH levothyroxine treatment is associated with favourable effects on, gestational age at delivery and early Caesarean section rates. Levothyroxine may also have some protective impact on stillbirths in SCH.

## O2 Pharmacological enhancement of radioiodine uptake using Src kinase inhibitors

### Vikki L. Poole, Alice Fletcher, Bhavika Modasia, Neil Sharma, Rebecca J. Thompson, Waraporn Imruetaicharoenchoke, Martin L. Read, Kristien Boelaert, Christopher J. McCabe, Vicki E. Smith

#### Institute of Metabolism and Systems Research, University of Birmingham, Birmingham, UK

##### **Correspondence:** Vicki E Smith (v.e.smith@bham.ac.uk)

**Background**

Radioiodine imaging, cell ablation and treatment of metastases in thyroid cancer rely on the presence of a functional sodium iodide symporter (NIS) in the basolateral plasma membrane (PM) of thyrocytes. Augmenting NIS PM localisation represents an important therapeutic strategy for increasing radioiodine delivery. We previously described a mechanism by which NIS is internalised by pituitary tumor-transforming gene-binding factor (PBF) in thyroid cells, significantly reducing radioiodine uptake. Importantly, we demonstrated that PBF phosphorylation at Y174 by Src kinase mediated NIS repression, which could be rescued by the Src family kinase (SFK) inhibitor PP1.

**Methods**

We have now replicated these findings in breast cancer cells, further elucidated the mechanism of repression and identified a more potent inhibitor of PBF-pY174.

**Results**

In MCF-7 and MDA-MB-231 breast cancer cells with either exogenous or all-trans retinoic acid/dexamethasone-induced NIS expression, PBF significantly repressed radioiodine uptake and this was reversible with PP1 treatment. PBF-Y174 is also a critical part of an endocytosis motif and mutation results in PM accumulation. Mutation of a predicted Src consensus sequence (EEN170-172AAA) abrogated pY174 and radioiodine uptake repression, confirming Src-dependent Y174 phosphorylation and demonstrating that NIS repression is mediated by phospho-PBF and not PM-bound PBF. Treatment with the SFK inhibitor dasatinib potently inhibited PBF-pY174 and restored radioiodine uptake. In the presence of dasatinib-resistant Src (T341I), dasatinib no longer rescued PBF repression of NIS, indicating that Src and no other SFK mediates PBF phosphorylation.

**Conclusions**

Taken together, these data suggest that Src inhibition can effectively enhance radioiodine uptake in multiple tumour types, with implications for improving outcomes in thyroid cancer and utilising NIS for the treatment of other tumours.

## O3 Comparative analysis of human and mouse expression data identifies proto-oncogene PTTG- and PBF-associated genes in thyroid cancer

### Martin L. Read^1^, Jim C. W. Fong^1^, Waraporn Imruetaicharoenchoke^1^, Bhavika Modasia^1^, Hannah Nieto^1^, Alice Fletcher^1^, Rebecca Thompson^1^, Neil Sharma^1^, John C. Watkinson^2^, Andrew S. Turnell^3^, Kristien Boelaert^1^, Vicki E. Smith^1^, Christopher J. McCabe^1^

#### ^1^Institute of Metabolism and Systems Research, University of Birmingham, Birmingham, UK; ^2^University Hospitals Birmingham National Health Service Foundation Trust, Birmingham, UK; ^3^Institute of Cancer and Genomic Sciences, University of Birmingham, Birmingham, UK

##### **Correspondence:** Martin L. Read (m.l.read.20@bham.ac.uk)

**Background**

Whilst the proto-oncogene PTTG and its binding partner PBF have been shown to be up-regulated in differentiated thyroid cancer, there is a paucity of information regarding their co-expression and specific roles in tumour progression. In particular, PTTG and PBF have both been reported to modulate the tumour suppressor p53, whose activity is impaired in most human cancers. Therefore, the role of PTTG and PBF in thyroid tumorigenesis may involve disruption of p53 pathways that are central to DNA-damage repair (DDR), cell growth and apoptosis.

**Methods**

In the present study we investigated the association of PTTG and PBF with p53-related genes in the TCGA thyroid cancer dataset, as well as in a bi-transgenic murine model (Bi-Tg) overexpressing PTTG and PBF specifically in the thyroid gland.

**Results**

Characterisation of primary murine Bi-Tg thyrocytes revealed that co-expression of PTTG and PBF caused extensive repression of DDR genes (39/82 genes; *P* < 0.05). Of these, 31 genes were down-regulated >1.5-fold, including genes with key roles in maintaining genomic integrity such as *Brca1*. Irradiation exposure to increase intracellular p53 further showed significant differences in overall DDR gene expression (n = 82 genes) between irradiated Bi-Tg and wild-type thyrocytes (*P* = 2.4 × 10^−4^) that was greater than either PBF-Tg (*P* = 1.5 × 10^−3^) or PTTG-Tg thyrocytes (*P* = NS). By comparison in the TCGA dataset, there were striking correlations with PTTG and PBF in well-characterised p53-related gene panels (*P* < 0.05; 82–96 genes per panel; n = 322 unmatched TCGA tumour samples). Importantly, nearly half of the significant DDR gene alterations in Bi-Tg thyrocytes were also present in TCGA comparing samples with either low or high PTTG/PBF mRNA levels. Furthermore, the overall survival (*P* = 0.0002) and disease-free survival (*P* = 0.02) was poorer for TCGA individuals with high tumoural PTTG/PBF expression (n = 20) than for all other patients (n = 255).

**Conclusions**

Altogether our findings provide important insights into the association of p53-related genes with PTTG and PBF in thyroid tumourigenesis.

## O4 Graves’ disease with fluctuating thyroid status and hypothyroidism with positive anti-TSH receptor antibody levels - distinctive autoimmune side-effects following alemtuzumab therapy for multiple sclerosis

### Nadia Pariani^1^, Mark Willis^2^, Ilaria Muller^3^, Sarah Healy^2^, Taha Nasser^2^, Joanne Jones^4^, Krishna Chatterjee^1^, Colin Dayan^3^, Neil Robertson^2^, Alasdair Coles^4^, Carla Moran^1^

#### ^1^Wellcome-MRC Institute of Metabolic Science, University of Cambridge, Cambridge, UK; ^2^Institute of Psychological Medicine and Clinical Neuroscience, Cardiff University & University Hospital of Wales, Cardiff, UK; ^3^Department & Institute of Molecular & Experimental Medicine, Cardiff University, Cardiff, UK ; ^4^Department of Clinical Neurosciences, University of Cambridge, Cambridge, UK

##### **Correspondence:** Carla Moran (cm682@medschl.cam.ac.uk)

**Background**

Alemtuzumab, a highly effective, newly-licensed, treatment for multiple sclerosis (MS), is associated with autoimmune side-effects - notably Graves’ disease (GD) with a reportedly indolent course.

**Aim**

To determine the type, frequency and course of thyroid dysfunction (TD) in a cohort of alemtuzumab-treated MS patients in Cambridge & Cardiff.

**Methods**

Case records of alemtuzumab-treated patients who developed TD were reviewed.

**Results**

Overall, 40% (102/249; 81 F, 21 M) of alemtuzumab-treated patients developed TD; 71 cases with follow up data (median 5 yrs; range 5–198 months) post TD were analysed. Mean TD onset was 23 months (range 2-107mths) following the most recent dose of alemtuzumab; most (89%) occurred within 3 years. GD, defined as hyperthyroidism with positive anti-TSH receptor antibody (TRAB) levels, occurred most commonly (47/71; 66%). 9 of these (20%) showed fluctuating thyroid status, (transitioning from hypo to hyperthyroidism or vice versa). 27 GD patients completed a course of anti-thyroid drug (ATD) therapy, with 48% (13/27) relapsing and 52% (14/27) in remission after drug withdrawal. 3 cases of thyroiditis and 7 cases of anti-TPO antibody positive hypothyroidism were identified; in 8 cases, hypothyroidism with negative anti-TPO but positive TRAB was recorded. In 5 thyrotoxic and 1 hypothyroid case, antibody testing is outstanding.

**Conclusion**

In our ascertained cohort, GD was the commonest cause of alemtuzumab-induced TD, with post therapy relapse being comparable to conventional GD. Fluctuating thyroid status in alemtuzumab-induced GD (20% of cases), together with an unexpectedly high occurrence of anti-TPO negative, TRAB-positive hypothyroidism, suggests that both stimulating and blocking anti-TSH receptor antibodies develop in this context.

## O5 Controlled Antenatal Thyroid Screening (CATS) Study II; (i) Effect of treatment of suboptimal gestational thyroid function (SGTF) on children’s behaviour at age 9

### Charlotte Hales^1^, Peter Taylor^1^, Sue Channon^2^, Kirsten McEwan^2^, Aled Rees^1^, John Gregory^1^, Ilaria Muller^1^, Mohd S Draman^1^, Colin Dayan^1^, Kate Langley^3^, Anita Thapar^3^, John Lazarus^1^ and Marian Ludgate^1^

#### ^1^Thyroid Research Group, Cardiff University School of Medicine, Cardiff, UK; ^2^Institute of Primary Care & Public Health, Cardiff University School of Medicine, Cardiff, UK; ^3^Institute of Psychological Medicine and Clinical Neurosciences, Cardiff University School of Medicine, Cardiff, UK

##### **Correspondence:** Charlotte Hales (halesc2@cardiff.ac.uk)

**Background**

Many studies have investigated the effects of maternal SGTF on cognition but little is known about its impact on behaviour. We addressed this question using mother/child pairs from the CATS study, at time of consent participants were randomised and some received levothyroxine therapy for SGTF from 12 weeks gestation. In CATS II, 120 from the treated and 106 from the untreated participated, as well as 244 from the normal thyroid function group (GTF).

**Methods**

When children were aged 9 behaviour was assessed by maternal reports using, The Strengths and Difficulties Questionnaire (SDQ), Child ADHD Questionnaire and Social Communication Questionnaire (SCQ); higher scores indicated more problems. Primary analysis used a MANCOVA, firstly with SGTF groups merged, and secondly by individual group. Secondary analysis explored fT4 during pregnancy and offspring behaviour; all analyses were Bonferroni corrected.

**Results**

The merged SGTF group had fewer peer problems (SDQ) (p = 0.008), but more ADHD overactivity problems (p = 0.020) than the normal GTF group. The analysis of the three groups revealed that treated SGTF scored higher than normal GTF (for ADHD overactivity p = 0.024) and the untreated SGTF (for SCQ, p = 0.047). ADHD overactivity was positively correlated to fT4 at six weeks post initiation of therapy. Children of over-treated mothers (fT4 > 17.7pmol/L) had higher scores for ADHD overactivity compared to the rest of the study group (p = 0.008).

**Conclusion**

Treatment of SGTF may exacerbate ADHD overactivity difficulties e.g. 11% of treated had scores >2SDs above the mean compared with 4% in normal and untreated. The analysis supports recent literature that SGTF over-treatment may have a negative effect and requires close monitoring throughout pregnancy.

## P1 Increased all-cause mortality, rehospitalisation rate and cardiovascular morbidity in hospitalised hyperthyroid patients – a nested case-control study

### Barbara Torlinska^1^, Jayne Franklyn^1^, Jamie Coleman^2^, Kristien Boelaert^1^

#### ^1^Institute of Metabolism and Systems Research, University of Birmingham, Birmingham, UK; ^2^Institute of Clinical Sciences, University of Birmingham, Birmingham, UK

##### **Correspondence:** Barbara Torlinska (b.torlinska@bham.ac.uk)

**Background**

Thyroid dysfunction (TD) often runs an indolent course and may remain undiagnosed for prolonged periods. Most subjects are treated as outpatients; effects of TD on hospitalised individuals are poorly studied. We set out to determine long-term influences of hyperthyroidism in a large cohort of inpatients evaluating their hospitalisation frequency, comorbidities and all-cause mortality.

**Methods**

A nested case-control study was conducted using a cohort admitted to a large tertiary centre (2007–2012). 671 hyperthyroid subjects were identified, matched (1:4) by age, gender and year of admission with hypo- and euthyroid inpatients and followed until 31/12/2015.

**Results**

A total of 31,400 person-years were analysed in 5,979 inpatients. There were 2,175 (36.4%) deaths. Hyperthyroidism was associated with a significant increase in all-cause mortality compared to hypo- and euthyroid inpatients (HR = 1.2 [95% CI:1.02–1.33], P = 0.03 and 1.3 [1.10–1.44], P = 0.001). Additionally, hyperthyroid inpatients were more frequently re-hospitalised (4.0 [3.5–4.5]) compared with controls in both groups (AOR_Ho_ = 2.1, P = 0.003; AOR_Eu_ = 4.8, P < 0.001). They were more frequently admitted for circulatory conditions (CVD) (N_Hr_ = 230, 34.3%; N_Ho_ = 711, 27.1%, AOR_Ho_ = 1.5 [1.2–1.8], P < 0.001; N_Eu_ = 601, 22.4%, AOR_Eu_ =1.9 [1.6–2.3], P < 0.001) while respiratory admissions were more common in hyperthyroid (N = 119, 17.7%) when compared with euthyroid (N = 357, 13.3%, AOR = 1.4 [1.1–1.8], P = 0.003) but not different from hypothyroid subjects; proportions of patients admitted for nervous and digestive causes were not significantly different. When considering recorded comorbidities, CVD was more frequent in hyperthyroid inpatients (N_Hr_ = 457, 68.1%) than matched controls (N_Ho_ = 1,679, 64.0%, AOR_Ho_ = 1.3 [1.1–1.6], P = 0.007; N_Eu_ = 1,442, 53.7%, AOR_Ho_ = 2.2 [1.8–2.8], P < 0.001) and presented more frequently with atrial fibrillation (N_Hr_ = 207, 30.8%; N_Ho_ = 441, 16.8%, AOR_Ho_ = 2.6 [2.1–3.2], P < 0.001; N_Eu_ = 339, 12.6%, AOR_Eu_ = 3.6 [2.9–4.5], P < 0.001) or heart failure (N_Hr_ = 105, 15.6%; AOR_Ho_ = 1.5 [1.2–1.9], P = 0.002; AOR_Eu_ = 2.6 [2.0–3.4], P < 0.001).

**Conclusion**

We conclude that hyperthyroidism in hospitalised patients is associated with additional health and economic burdens, significantly increasing likelihood of re-hospitalisation, cardiovascular morbidity and all-cause mortality.

## P2 Controlled Antenatal Thyroid Screening (CATS) Study II; (ii) Effect of treatment of suboptimal gestation thyroid function (SGTF) on children’s cognition at age 9

### Charlotte Hales^1^, Peter Taylor^1^, Sue Channon^2^, Kirsten McEwan^2^, Lei Zhang^1^, Ameen Bakhsh^1^, Michael Gyedu^1^, Aled Rees^1^, Onyebuchi Okosieme^1^, John Gregory^1^, Ilaria Muller^1^, Mohd S Draman^1^, Colin Dayan^1^, John Lazarus^1^, Marian Ludgate^1^

#### ^1^Thyroid Research Group, Cardiff University School of Medicine, Cardiff, UK; ^2^Institute of Primary Care & Public Health, Cardiff University School of Medicine, Cardiff, UK

##### **Correspondence:** Charlotte Hales (halesc2@cardiff.ac.uk)

**Background**

The Controlled Antenatal Thyroid Screening (CATS) study was the first randomized controlled trial to investigate the effect of antenatal thyroid screening and maternal levothyroxine treatment on childhood cognition, evaluated at 3 years of age. Although CATS did not show treatment benefits, the childhood assessment may have been undertaken too early for differences to be apparent.

**Methods**

In the present study cognition was re-assessed in offspring of the Welsh CATS cohort aged 9 years using the WISC-IV (IQ). Groups comprised children of mothers with normal gestational thyroid function (GTF, n = 233) and those of mothers with treated (n = 118) and untreated (n = 101) SGTF, i.e. TSH in the highest 2.5% and/or fT4 in the lowest 2.5%. Analysis included a regression to explore the odds of IQ < 85 (1SD below the mean). A secondary analysis explored children’s Thr92Ala polymorphism and this was compared to maternal thyroid status.

**Results**

There was no difference in proportion of children with IQ < 85 between children of women with SGTF (10%) and normal GTF (6.4%) (p = 0.59) or between treated (8.5%) and untreated (11.9%) SGTF results (p = 0.29). Children homozygous for Thr92Ala and born to mothers with low maternal fT4 levels, indicated a trend for IQ < 85 compared to the normal GTF group: OR = 2.90 (95%CI 0.49,17.30) p = 0.24. Removing the treated group increased the odds of IQ < 85: OR = 13.7 (95%CI 1.63,115) p = 0.01.

**Conclusion**

Our results do not support a beneficial effect of antenatal thyroid screening on child cognition. One in seven of the population have the Ala92Ala genotype and are more at risk of adverse effects of SGTF. Further research is needed to clarify the impact of the Thr92Ala polymorphism and its interaction with maternal thyroid function on child cognition.

## P3 Genetic variants associated with hypothyroidism and serum thyroid stimulating hormone levels (TSH) in Tayside (Scotland): a GoDARTS study

### Enrique Soto-Pedre^1^, Graham P. Leese^2^, Kaixin Zhou^1^

#### ^1^Division of Molecular and Clinical Medicine, School of Medicine, Ninewells Hospital and Medical School, University of Dundee, Dundee, Scotland, UK; ^2^Department of Endocrinology and Diabetes, Ninewells Hospital and Medical School, University of Dundee, Dundee, Scotland, UK

##### **Correspondence:** Enrique Soto-Pedre (e.soto@dundee.ac.uk)

**Background**

Hypothyroidism is the most common thyroid disorder. Genome-wide association studies (GWAS) have identified variants in FOXE1 that are associated with hypothyroidism, and PDE8B and CAPZB that are associated with TSH levels. However, the number of studies is limited and replication studies are desirable.

**Methods**

Patients were identified from a study of the Genetics of Diabetes Audit and Research Tayside (GoDARTS) recruited in Tayside, Scotland, between January 1996 and March 2014. Electronic Medical records (EMR)-derived phenotypes were ascertained and single-locus tests of association were performed via logistic regression under the assumption of an additive genetic model. Serum TSH levels were identified in 16,769 patients of white ethnicity, 2,664 with hypothyroidism and 14,105 controls.

**Results**

A FOXE1 variant (rs925489) was associated with hypothyroidism (OR = 0.77 [95%CI 0.71–0.83], P = 1.9 × 10^−10^). We found evidence that other genetic variants previously associated with hypothyroidism were also associated in this study. We found association with PTPN22 (rs6679677, P = 3.0 × 10^−04^), SH2B3 (rs3184504, P = 9.4 × 10^−05^), VAV3 (rs4915077, P = 9.9 × 10^−03^), and HLA (rs2517532, P = 7.5 × 10^−05^). The analysis found the association of PDE8B (rs4704397, P = 1.0 × 10^−22^; rs6885099, P = 1.1 × 10^−32^) and CAPZB (rs10799824; P = 8.2 × 10^−12^) with serum TSH levels in euthyroid individuals. These genetic variants explained 1.67, 1.57, and 0.82% of the variation of mean TSH levels respectively.

**Conclusions**

Our results replicated the previously reported association of FOXE1 with hypothyroidism, and PDE8B and CAPZB with serum TSH levels, and emphasize the need for additional genetic studies in more diverse population.

## P4 A novel ESR2 frameshift mutation predisposes to medullary thyroid carcinoma and causes inappropriate RET expression

### Martin L. Read^1^, Joel A. Smith^1^, Jon Hoffman^2^, Vicki E. Smith^1^, Neil V. Morgan^3^, Christopher Campbell^2^, Naomi C. Wake^1^, John C. Watkinson^4^, Yvonne Wallis^5^, Eamonn R. Maher^1,6^, Christopher J. McCabe^1^, Emma R. Woodward^1,7^

#### ^1^Institute of Metabolism and Systems Research, University of Birmingham, Birmingham, UK; ^2^West Midlands Regional Genetics Service, Birmingham Women’s Hospital, Birmingham, UK; ^3^Institute of Cardiovascular Sciences, University of Birmingham, Birmingham, UK; ^4^University Hospitals Birmingham National Health Service Foundation Trust, Birmingham, UK; ^5^West Midlands Regional Genetics Laboratory, Birmingham Women’s Hospital, Birmingham, UK; ^6^Department of Medical Genetics, University of Cambridge, Cambridge, UK; ^7^Manchester Centre for Genomic Medicine, St. Mary’s Hospital, Manchester, UK

##### **Correspondence:** Martin L. Read (m.l.read.20@bham.ac.uk)

**Background**

Familial medullary thyroid cancer (MTC) is associated with germline RET mutations causing multiple endocrine neoplasia type 2 (MEN2). The majority of germline RET mutations predisposing to MEN2 result in single amino acid substitutions causing inappropriate constitutive RET activation. However, some rare families with apparent MTC predisposition do not have a detectable RET mutation (non-RET), suggesting that further predisposing gene alterations remain to be identified.

**Methods**

Here, we undertook exome sequencing studies in one family with apparent predisposition to non-ret MTC and identified a novel ESR2 frameshift mutation, c.948delT; p.G318Afs*22, which segregated with histological diagnosis following thyroid surgery. Sequencing of ESR2 in individuals with apparently isolated MTC identified a further novel germline missense alteration, c.382G > C; p.V128L in a female who developed MTC age 36 years.

**Results**

Functional assays showed that ESR2-V128L retained transcriptional activity with a significant increase in luciferase activity in response to ESR2-agonist DPN in HCT116 (3.7-fold; *P* < 0.01) and MCF-7 (1.8-fold; *P* < 0.05) cells. In contrast, ESR2-G318Afs*22 was incapable of inducing luciferase activity in either cell line (*P* = NS). Furthermore, ESR2-G318Afs*22 failed to inhibit ESR1 driven luciferase activity in response to either 17β-estradiol (E2) or ESR1-agonist PPT, or restrain ESR1-driven proliferation of MCF-7 cells. In contrast, wild-type (WT) ESR2 and ESR2-V128L inhibited ESR1-driven luciferase activity (>60%. *P* < 0.01) and cell proliferation (>30%, *P* < 0.05). As RET expression is known to be stimulated by oestrogen, we then determined the influence of ESR2 mutants on RET in E2- and PPT-treated HCT116 cells. In contrast to WT ESR2, ESR2-G318Afs*22 was unable to oppose ESR1-stimulation of the RET proto-oncogene at both the mRNA and protein level. Treatment with 4-hydroxytamoxifen was however capable of inhibiting E2-induced RET mRNA expression in cells with ESR2-G318Afs*22.

**Conclusions**

Together these data indicate an emerging role for ESR2 as a novel susceptibility gene in non-RET MTC development, especially as ESR2-G318Afs*22 was associated with elevated RET.

## P5 Use of TSH-Receptor Antibodies (TRAb) in the assessment of new onset thyrotoxicosis within the North East of England

### Su A. Tee^1^, David R. Bishop^2^, Ahmed Al-Sharefi^3^, Owain Leng^4^, Robert A. James^5^

#### ^1^Department of Diabetes and Endocrinology, Northumbria Healthcare NHS Foundation Trust, Newcastle, UK; ^2^Department of Diabetes and Endocrinology, Sunderland Royal Hospital, Sunderland, UK; ^3^Department of Diabetes and Endocrinology, Darlington Memorial Hospital, Darlington, UK; ^4^Department of Diabetes and Endocrinology, South Tyneside General Hospital, South Tyneside, UK; ^5^Department of Endocrinology, Royal Victoria Infirmary, Newcastle, UK

##### **Correspondence:** Su A Tee (tsun83@gmail.com)

**Background**

Causes of hyperthyroidism include Graves’ disease (GD), toxic multinodular goitre and thyroiditis. British Thyroid Association guidelines recommend specialist referral once hyperthyroidism is diagnosed. TSH-receptor antibodies (TRAb) are more specific to GD than thyroid peroxidase antibodies (TPOAb). Regional guidelines recommend measuring TRAb in thyrotoxic patients. We aimed to assess TRAb use regionally in newly diagnosed thyrotoxicosis.

**Method**

We conducted a retrospective case review of patients with newly detected thyrotoxicosis from 1^st^ March-31^st^ August 2015 from 4 endocrinology centres. TSH, fT4, fT3, TPOAb and TRAb values; the requester and date requested were recorded. Any thyroid uptake scans done; and final diagnoses were noted.

**Results**

We analysed 209 records – 79% females (n = 166), 21% males (n = 43); the average age was 50.5 (17–96). 88.6% had TRAb requested. 76.2% requests were from endocrinologists, 15.7% from GPs, 5.9% from Biochemistry, and 2.2% from other physicians. 56.2% had positive TRAbs. The commonest diagnosis was GD (55.0%), followed by multinodular goitre (10.5%) and thyroiditis (8.1%). GD was diagnosed using TRAb in 81.7%; and using thyroid uptake scans in 6% (n = 7). 2 patients had clinical features; in 12 patients, the reason for favouring a GD diagnosis was unclear. 19 patients (9%) were not referred to endocrinology. Average time between detection of thyrotoxicosis and TRAb request was 40 days (32–57 days). Time to TRAb request was shorter (average 9 days) if requested prior to referral to an endocrinologist. This lag was because most TRAb requests were only requested after referrals were received and reviewed by endocrinologists.

**Conclusions**

TRAb is commonly used regionally with good sensitivity and specificity. We propose that it should be added on by biochemistry labs in all newly thyrotoxic patients, as this could expedite diagnosis, minimising use of inappropriate antithyroid medications. Availability of results before endocrinology consultations would also facilitate prompt treatment and better communication with patients.

## P6 Optimising the thyroxine absorption test in patients with persistently raised TSH

### Rashim Salota^1^, Andrew Rodin^2^, Nikhil Johri^1^, Stephen L. Hyer^2^

#### ^1^Chemical Pathology, Epsom and St Helier Hospitals NHS Trust, Carshalton SM5 1AA, UK; ^2^Departments of Endocrinology, Epsom and St Helier Hospitals NHS Trust, Carshalton SM5 1AA, UK

##### **Correspondence:** Rashim Salota (rashim.salota@esth.nhs.uk)

**Background**

The thyroxine absorption test is an established test to investigate the cause of persistently raised TSH in patients on levothyroxine. We review our experience with a modified form of this test in our clinical setting.

**Method**

Patients with persistently raised TSH despite adequate thyroxine dose and admitting good compliance were offered the test. They were advised to fast and bring their own medication. A recent ECG, medical history (including medications) was reviewed. Bloods were taken for a baseline FT4, FT3 and TSH along with vitamin B12, folate, ferritin, iron, transferrin, vitamin D, SHBG, coeliac screen and gastric parietal antibodies.

A single (weekly) dose of levothyroxine calculated as [1.6 × weight (kg) × 7 μg] was given orally under direct observation and FT4 and TSH re-measured at 2 hours. Thereafter FT4 and TSH were measured weekly (trough) and levothyroxine administered under observation.

An increase in FT4 at 2 hours by ≥50% following initial dose indicated adequate GI absorption. Test was terminated once TSH fell within the normal range. In patients where TSH was not at target despite evidence of adequate absorption at 4 weeks, the levothyroxine dose was increased to [2.0 × weight (kg) × 7 μg] weekly for 2 weeks; if TSH still remained elevated; the same dose was divided and given as twice weekly for another 2 weeks. TFT results after 6 months or more were collected on the patients to check the effect of thyroxine absorption test on long term compliance.

## Results

Fourteen patients (two male, twelve female) with a mean age of 41 years (16–74), and weight 89 kg (53–124) underwent the test. Cause of hypothyroidism was autoimmune in 8, total thyroidectomy in 5 and congenital hypothyroidism in 1. Before testing, mean daily levothyroxine dose was 209 μg/day (range: 100–300). In 9 there was adequate absorption of levothyroxine and TSH normalised in 4 weeks confirming poor compliance. In one patient TSH normalised after receiving the higher dose while two patients required higher amount in divided dose to normalise TSH. One patient on continuous ambulatory peritoneal dialysis had inadequate FT4 response and failed to normalise TSH at 8 weeks. TSH however normalised following renal transplantation. Malabsorption due to total pancreatectomy was the cause of TSH non-suppression in one. TFT results available on 10 patients, 6 months or more after the test showed that 70% of the patients were maintaining TSH within normal range.

## Conclusion

Thyroxine absorption test was useful in differentiating poor compliance from other causes of persistently raised TSH. Some patients who fail to suppress TSH after 4 weeks may require a higher dose of levothyroxine administered once or twice weekly. Splitting dose as twice weekly may be useful as in some individuals as t1/2 may be shorter. Offering continuation as once or twice weekly dose may help improve compliance over long term.

## P7 Diagnosis and workup of thyrotoxicosis – a secondary care audit

### Thomas Mcdonnell, Cheong G Ooi

#### Department of Diabetes and Endocrinology, Aintree University Hospital, Liverpool, UK

##### **Correspondence:** Thomas Mcdonnell (tm9038.2009@my.bristol.ac.uk)

**Background**

Identification of the cause of thyrotoxicosis is important as this translates to different treatments and prognosis. Using the current NICE clinical knowledge summary (CKS), BTA guidelines for the use of thyroid function tests, and a recent clinical review by B Vaidya *et al (*2014), audit standards for appropriate diagnosis of thyrotoxicosis were established.

**Methods**

All new patients at the hospital thyroid clinic in January 2015 were screened. Data was gathered from referral letters, clinic notes, and hospital information system. 37 patients were eligible.

**Results**

Treatment was initiated by the GP at the time of diagnosis in 13 of the 37 (35.1%). 19 patients had thyroid antibodies checked (51.35%) however only 1 of the 19 had TRAb included (5.55%). At initial endocrine OPA 32 of 36 had their TRAb checked (88.9%) and 3 of 35 (8.5%) had an RIUS or USS ordered.

**Conclusions**

51% of GPs ordered thyroid antibodies, but only 1 patient had TRAb requested, suggesting that GPs were not aware of the benefit of measuring TRAb (as the diagnostic hallmark of Graves) or did not have access to this test. The NICE CKS does not ask the primary care physician to measure TRAb, although most clinical experts suggest its measurement on diagnosis of thyrotoxicosis. As TRAb was usually only ordered at the first outpatient appointment, only 8% of patients had RIUS/USS ordered at their first hospital appointment. 35% of patients had carbimazole initiated by GPs. This has implications for diagnosis and long-term management, especially in the absence of appropriate antibody testing and mean time from referral to first appointment of 35 days. A proposed change in our pathway is the automatic addition of TRAb testing in all newly detected cases of thyrotoxicosis when noted by our laboratory.

## P8 Iodine deficiency amongst pregnant women in South-West England

### Bridget A. Knight^1, 2^, Beverley M. Shields^1^, Elizabeth Pearce^3^, Lewis Braverman^3^, Xuemei He^3^, Rachel Sturley^4^, Bijay Vaidya^5^

#### ^1^NIHR Exeter Clinical Research Facility, University of Exeter Medical School, Exeter, UK; ^2^R&D Department, Royal Devon and Exeter NHS Foundation Trust, Exeter, UK; ^3^Section of Endocrinology, Diabetes & Nutrition, Boston University School of Medicine, Boston, MA, USA; ^4^Department of Women’s Health, Royal Devon and Exeter NHS Foundation Trust, Exeter, UK; ^5^Department of Endocrinology, Royal Devon and Exeter NHS Foundation Trust, Exeter, UK

##### **Correspondence:** Bijay Vaidya (B.Vaidya@exeter.ac.uk)

**Background**

Iodine is important for thyroid hormone synthesis, and iodine deficiency in pregnancy may impair foetal neurological development. The UK population is generally thought to be iodine sufficient; however recent studies have questioned this assumption. The WHO recommends the optimum median urinary iodine concentrations (UIC) as 150-249 μg/l in pregnancy. Our study aimed to explore the prevalence of iodine deficiency in a cohort of pregnant mothers from South-West England.

**Methods**

Urine samples were obtained from 308 women participating in a study of breech presentation in late pregnancy. They had no known thyroid disease and a singleton pregnancy at 36–38 weeks gestation. Samples were analysed for UIC. Baseline data included: age, parity, smoking status, ethnicity, BMI at booking, vitamin use, and validated dietary questionnaire. There was no difference in median UIC between women with (n = 156) or without (n = 152) a breech presentation (p = 0.8) so subsequent analyses were carried out as a combined group.

**Results**

Participants had a mean (SD) age 31(5) years, median (IQR) BMI 24.4(22.0, 28.3) kg/m^2^, 41% were primiparous, 10% smoked during pregnancy, 34% took iodine (>140 μg/pill) containing vitamins. 96% were Caucasian. Median (IQR) UIC was 88.0 (54.1, 157.5) μg/l, which is consistent with iodine deficiency by WHO criteria. A total of 224/308 (73%) of women had UIC values <150 μg/l. Increasing milk intake was associated with higher UIC (p = 0.003). There was no difference in median (IQR) UIC between those women who took iodine containing vitamins (n = 106) and those who did not (n = 202): 88.5(54.5, 170.5) vs 88.0 (53.8, 150.0) μg/l, p = 0.6. There was no correlation between median UIC and TSH (p = 0.6) or FT4 (p = 0.1).

**Conclusion**

Iodine deficiency in pregnancy is common in South-west England. Further research is needed to develop optimum prevention and treatment strategies.

